# Nanoarchitectonics on Z-scheme and Mott–Schottky heterostructure for photocatalytic water oxidation *via* dual-cascade charge-transfer pathways†

**DOI:** 10.1039/d3na00182b

**Published:** 2023-05-10

**Authors:** Yao Li, Siyuan Liu, Runlu Liu, Jian Pan, Xin Li, Jianyu Zhang, Xiaoxiao Zhang, Yixin Zhao, Dawei Wang, Hengdao Quan, Shenmin Zhu

**Affiliations:** a State Key Laboratory of Metal Matrix Composites, School of Materials Science and Engineering, Shanghai Jiao Tong University Shanghai 200240 P. R. China smzhu@sjtu.edu.cn; b Particles and Catalysis Research Group, School of Chemical Engineering, University of New South Wales Sydney 2052 Australia; c School of Environmental Science and Engineering, Shanghai Jiao Tong University Shanghai 200240 China; d School of Chemical Engineering and Environment, Beijing Institute of Technology Beijing 100081 China

## Abstract

The bottleneck for water splitting to generate hydrogen fuel is the sluggish oxidation of water. Even though the monoclinic-BiVO_4_ (m-BiVO_4_)-based heterostructure has been widely applied for water oxidation, carrier recombination on dual surfaces of the m-BiVO_4_ component have not been fully resolved by a single heterojunction. Inspired by natural photosynthesis, we established an m-BiVO_4_/carbon nitride (C_3_N_4_) Z-scheme heterostructure based on the m-BiVO_4_/reduced graphene oxide (rGO) Mott–Schottky heterostructure, constructing the face-contact C_3_N_4_/m-BiVO_4_/rGO (CNBG) ternary composite to remove excessive surface recombination during water oxidation. The rGO can accumulate photogenerated electrons from m-BiVO_4_ through a high conductivity region over the heterointerface, with the electrons then prone to diffuse along a highly conductive carbon network. In an internal electric field at the heterointerface of m-BiVO_4_/C_3_N_4_, the low-energy electrons and holes are rapidly consumed under irradiation. Therefore, spatial separation of electron–hole pairs occurs, and strong redox potentials are maintained by the Z-scheme electron transfer. These advantages endow the CNBG ternary composite with over 193% growth in O_2_ yield, and a remarkable rise in ·OH and ·O_2_^−^ radicals, compared to the m-BiVO_4_/rGO binary composite. This work shows a novel perspective for rationally integrating Z-scheme and Mott–Schottky heterostructures in the water oxidation reaction.

## Abstract 2

A face-contact C_3_N_4_/m-BiVO_4_/rGO ternary composite was constructed for excessive surface recombination during water oxidation. Spatial separation of electron–hole pairs occurs, and strong redox potentials are maintained by the Z-scheme electron transfer.

## Graphical abstract

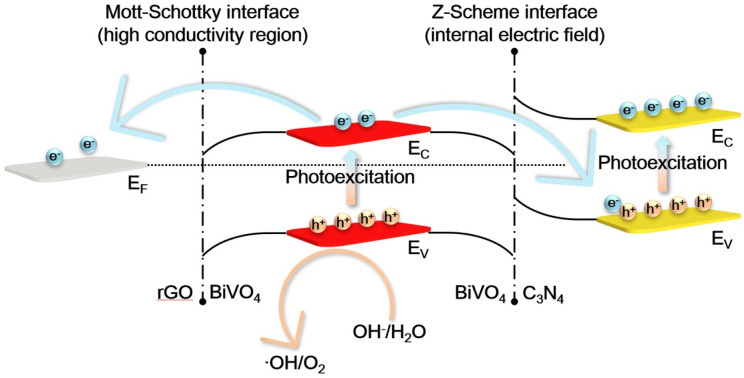

## Introduction

Since pressing energy and environmental issues are afflicting modern society, humans depend more and more on new sources of renewable energy.^1^ To this end, converting inexhaustible solar energy into chemical bonds through artificial photosynthesis, *e.g.*, water reduction into hydrogen fuel, is desirable for energy storage.^2^ Along with the process of photocatalytic water reduction, photocatalytic water oxidation (PWO) determines the overall reaction rate of water splitting. As a thermodynamically uphill process, the PWO reaction is a four-proton/four-electron step with sluggish kinetics, involving the dissociation of O–H chemical bonds as well as the formation of O–O chemical bonds.^3,4^ Targeting high-efficiency PWO, monoclinic bismuth vanadate (m-BiVO_4_) has stood out as one of the finest PWO catalysts since Kudo's report in 1999.^5^ To date, m-BiVO_4_ has been extensively studied due to its lone-pair effect, local electric field, appropriate band structure, earth abundance, and low toxicity.^6–8^ Despite vigorous development of single m-BiVO_4_, its PWO performance still falls short of expectations, owing to hampered carrier transfer and excessive carrier recombination, particularly on the surface and/or interface.^9^ Among the booming examples of modified tactics, heterointerface charge modulation has proven to be a useful method to elevate charge separation within photocatalysts.^10^ Nevertheless, there is still a lot of room to reconstruct m-BiVO_4_-based composites *via* heterojunction engineering,^11^ which should fine-tune the surface and/or interface properties with regard to the PWO reaction.

The Mott–Schottky heterostructure constructed by coupling semiconductors and reduced graphene oxide (rGO) is supposed to exert the distinct advantages of each component.^12,13^ Meanwhile, the heterointerface between adjacent components works for charge transfer and separation. With regard to m-BiVO_4_, rGO possessing surface oxygen-containing groups (OCGs) offers the prerequisites for anchoring m-BiVO_4_. Upon intimate coupling, the rGO component can darken the composite for light absorption, speed up electron transfer for high conductivity, and supply abundant active sites with large specific surface areas.^14^ Significantly, a high conductivity region at the heterointerface of m-BiVO_4_/rGO ensures spontaneous electron transfer over the Mott–Schottky heterojunction.^15,16^ Therefore, when the m-BiVO_4_ component enters into the excited state, its photogenerated electrons are conducive to flow toward rGO and can diffuse along a carbon network. Even though heterointerface charge modulation is implemented in the current m-BiVO_4_/rGO Mott–Schottky heterostructure, the m-BiVO_4_ component with a two-dimensional (2D) layered morphology in fact possesses another bare surface, which is not in contact with the rGO component. In this context, some photogenerated charge carriers still undergo surface recombination on the m-BiVO_4_ component, while others migrate to the heterointerface between m-BiVO_4_ and rGO.^17,18^ Therefore, without hindering the existing interfacial interaction and electron transfer in the m-BiVO_4_/rGO Mott–Schottky heterostructure, how to further overcome this surface recombination on the m-BiVO_4_ component remains challenging.

Green plants are known to convert CO_2_ and H_2_O into O_2_ and carbohydrates through natural photosynthesis, whereby photogenerated electrons are transported under a Z-scheme mode.^19^ Mimicking natural photosynthesis to build an artificial Z-scheme heterostructure is an optimal route to suppress surface recombination on a single semiconductor.^20,21^ The carbon nitride (C_3_N_4_) candidate, as a typical 2D photocatalytic material, belongs to a redox-complementary component for the direct Z-scheme heterostructure.^22–25^ Once the m-BiVO_4_/rGO (BG) Mott–Schottky heterostructure has been acquired, further coupling of C_3_N_4_ with the m-BiVO_4_ component may additionally construct the m-BiVO_4_/C_3_N_4_ Z-scheme heterostructure. Between m-BiVO_4_ and C_3_N_4_, the 2D/2D face-to-face contact heterointerface featuring an effective charge transfer is a promising candidate to tackle surface recombination on the m-BiVO_4_ component.^26^ However, so far, no studies have established a Z-scheme heterostructure based on a Mott–Schottky heterostructure. Given that one surface of m-BiVO_4_ is in contact with rGO to form a heterointerface, the other bare surface of m-BiVO_4_ provides an opportunity to bear the m-BiVO_4_/C_3_N_4_ Z-scheme heterostructure. This additional 2D/2D intimate coupling can be predicted according to the large contact area, limited crystal boundary, and strong electronic coupling effect between the dimension-matched 2D layered materials.^27,28^ Profiting from the synergistic role of the Mott–Schottky and Z-scheme heterostructure, especially the spatial separation of carriers and the sustained high redox potentials of components in the latter, the C_3_N_4_/m-BiVO_4_/rGO (CNBG) ternary composite with face-to-face contact is speculated to achieve excellent PWO performance.

Herein, using the m-BiVO_4_/rGO Mott–Schottky heterostructure as a prototype, we also established the m-BiVO_4_/C_3_N_4_ Z-scheme heterostructure. The obtained CNBG ternary composite with face-to-face contact is postulated to solve the excessive surface recombination during the PWO reaction. To study the synergistic role of this dual heterostructure, particularly the virtues of Z-scheme electron transfer, extensive investigation of physical structures, chemical status, optoelectrical features, and catalytic performance was performed, and the related PWO mechanism is further illustrated. As expected, the CNBG ternary composite shows over 193% growth in O_2_ yield compared with the binary composite and this PWO performance boost is validated by the increase in reactive oxygen species.

## Results and discussion

CNBG was fabricated using a multi-step process. In detail, the pristine C_3_N_4_ was synthesized *via* thermal polymerization from pre-heated melamine and chloride precursors, and the BG binary composite was obtained *via* hydrothermal treatment. The CNBG ternary composite was synthesized by simply mixing C_3_N_4_ and BG in deionized water followed by freeze-drying. To investigate the formation of CNBG, the zeta potentials of pristine C_3_N_4_, BG, and CNBG were measured in aqueous solution (Fig. S1†). The decrease in values from −8.7 mV for BG to −30.9 mV for CNBG is due to the incorporated C_3_N_4_ (−41.5 mV), certifying that the CNBG ternary composite was smoothly obtained based on the BG binary composite.

**Fig. 1:**
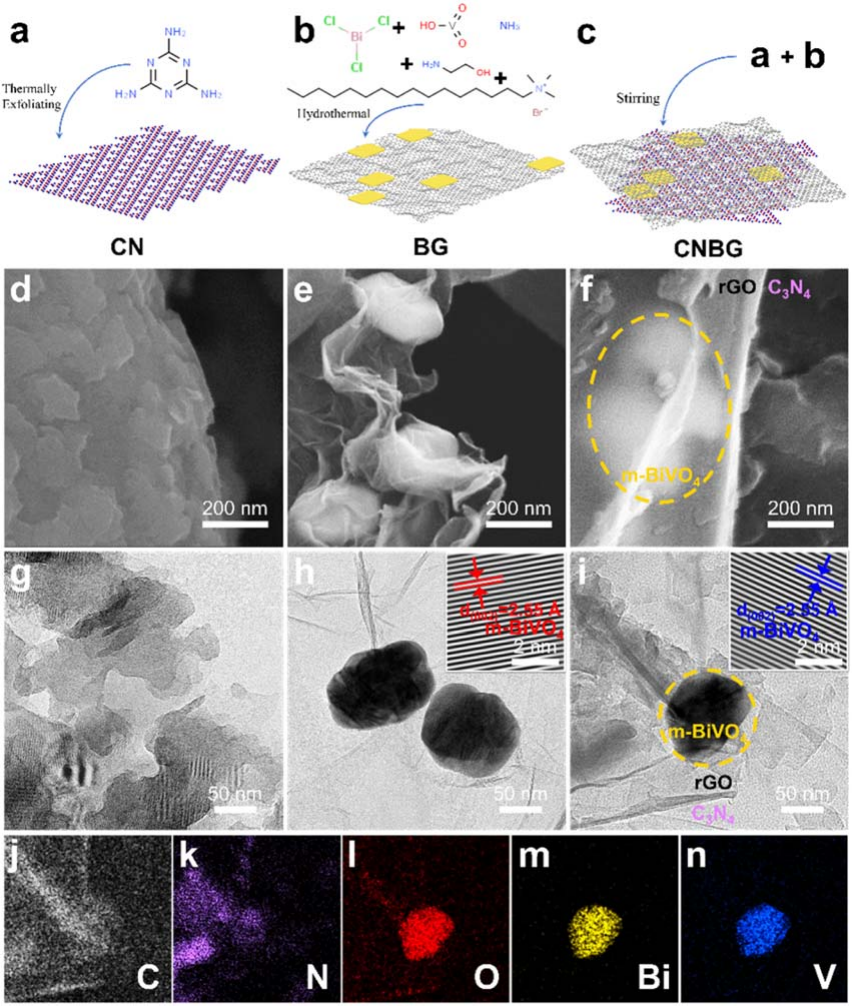
Characterization of the physical structures of C_3_N_4_, BG, and CNBG. Microstructure models and fabrication of (a) C_3_N_4_, (b) BG, and (c) CNBG. SEM images of (d) C_3_N_4_, (e) BG, and (f) CNBG. TEM images of (g) C_3_N_4_, (h) BG, and (i) CNBG. Inset: HRTEM images. (j–n) Corresponding elemental mapping images of CNBG in (i).

The physical structures were unveiled by microstructure models (Fig. 1a–c), scanning electron microscopy (SEM), and transmission electron microscopy (TEM) images (Fig. 1d–i). The pristine C_3_N_4_ shows irregular, lamellar-like, and stacking layers with a rough surface (Fig. 1d and g), and these structural characteristics accord with C_3_N_4_ reported previously.^29,30^ In terms of the BG binary composite, the sheet-shaped BiVO_4_ is uniformly distributed on rGO, the size of the BiVO_4_ nanosheets exceeds 100 nm, and the surface of rGO seems wrinkled and smooth (Fig. 1e and h). After the incorporation of C_3_N_4_ in the BG binary composite, the BG is covered by C_3_N_4_ to form the CNBG ternary composite, which is like a “sandwich”—the top layer is the smooth rGO with translucence under electron beam imaging, the bottom layer is the C_3_N_4_ with a rough surface, and the m-BiVO_4_ nanosheets with size over 100 nm are co-wrapped between the top and bottom layers, in perspective view (Fig. 1f and i). The face-to-face contact of the three components in CNBG, in particular, is desirable for heterointerface electron transfer, whereas their similar Brunauer–Emmett–Teller (BET) specific surface areas imply that the incorporated C_3_N_4_ makes no difference to the overall surface area of CNBG (Fig. S2†).

Elemental mapping images show the homogeneous distribution of C, N, O, Bi, and V elements across the CNBG (Fig. 1j–n). The existing areas of C and N elements are consistent with C_3_N_4_, the shapes of the Bi, O, and V elements are identical to m-BiVO_4_ nanosheets, and the partial regions of C and O elements can be attributed to rGO. These images corroborate a ternary hybrid architecture, namely the m-BiVO_4_ co-wrapped by C_3_N_4_ and rGO with intimate contact. The characterization of BG and CNBG from high-resolution TEM (HRTEM) shows an interplanar spacing of 2.55 Å for the (002) spacing of m-BiVO_4_ (insets in Fig. 1h and i), while the lattice fringes of C_3_N_4_ cannot be detected due to the poor crystallinity. Therefore, the incorporation of C_3_N_4_ shows no impact on the morphology and crystal structure of m-BiVO_4_.

**Fig. 2:**
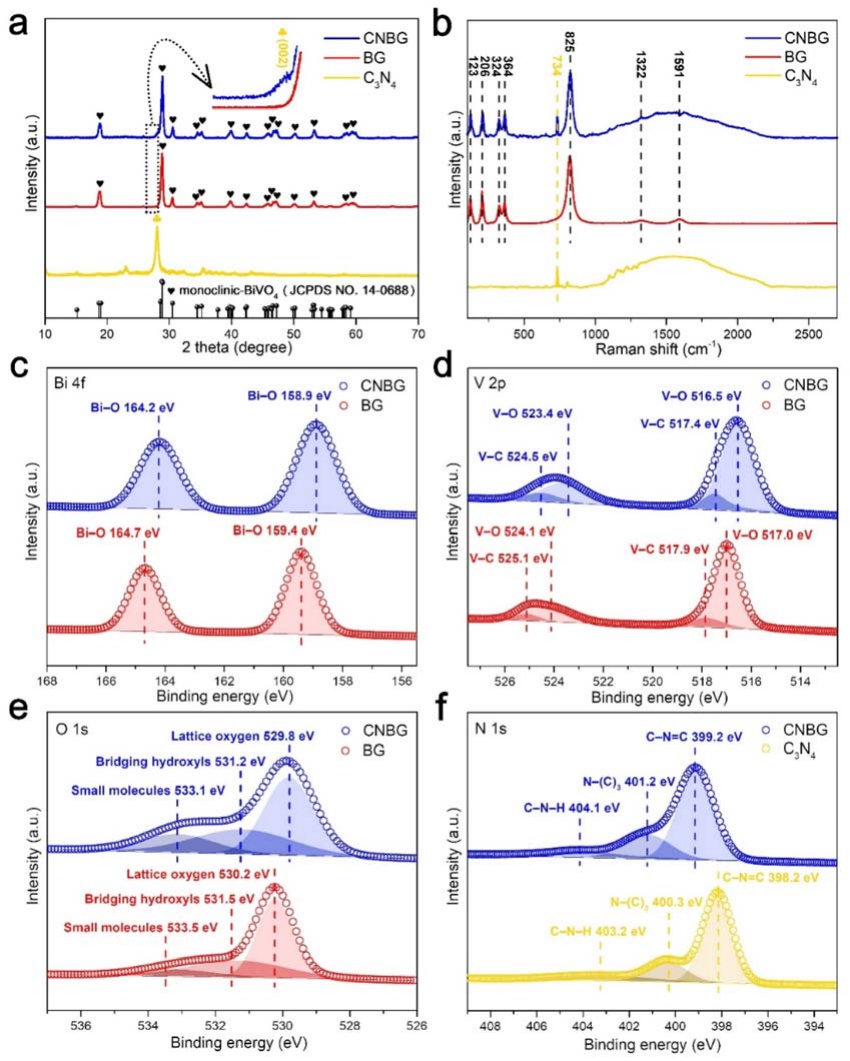
Characterization of the chemical status for the C_3_N_4_, BG, and CNBG. (a) XRD patterns. (b) Raman spectra. High-resolution XPS spectra of (c) Bi 4f, (d) V 2p, (e) O 1s, and (f) N 1s.

To further reveal the crystal structure information, X-ray diffraction (XRD) peaks for BG and CNBG, except for a peak at 27.8°, are both indexed to the m-BiVO_4_ (Fig. 2a), and their Raman bands (100 to 1000 cm^−1^), except for a peak at 734 cm^−1^, are also assigned to m-BiVO_4_ (Fig. 2b). These results agree with the HRTEM images and validate the m-BiVO_4_ being highly crystalline and phase pure, excluding any impact from the incorporated C_3_N_4_ on the crystal structure of m-BiVO_4_. Distinct from the BG binary composite, the CNBG ternary composite shows a small peak for the C_3_N_4_ (002) plane at 27.8° (in Fig. 2a), which belongs to the interlayer stacking of the conjugated aromatic segments.^31^ However, this peak in CNBG is much weaker than that in pristine C_3_N_4_, indicating decreased crystallinity within the C_3_N_4_ framework after its incorporation. A similar phenomenon is apparent in the Raman spectra (Fig. 2b), where the CNBG ternary composite presents a spike at 734 cm^−1^ and a broad peak (1000 to 2250 cm^−1^) relative to the BG, which are consistent with in-plane bending and disordered graphitic carbon–nitrogen vibrations, respectively .^32^ These additional peaks also emerge for pristine C_3_N_4_, suggesting the structure of C_3_N_4_ is preserved in CNBG after its incorporation. All these findings unambiguously reflect that the CNBG ternary composite was subtly constructed, and the sandwich-like structure with face-to-face contact is potentially useful for photocatalytic reactions.

To glean precise information on the heterointerface between m-BiVO_4_ and C_3_N_4_ in CNBG, the surface chemical status was analyzed by X-ray photoelectron spectroscopy (XPS). The change in binding energy reflects the interfacial chemical interaction and the different electron densities in the semiconductor–semiconductor heterojunction. The electron density increases when the binding energy shifts negatively, and decreases when the binding energy shifts positively.^33^ As shown in Fig. 2c, the Bi 4f spectrum of CNBG shows two peaks at 164.2 and 158.9 eV assigned to the binding energies of Bi–O chemical bonds; however, these peaks are negatively shifted compared with BG. Parallel negative shifts can be found in V 2p and O 1s spectra as well; the binding energies of the V–O and V–C chemical bonds appear at 523.4 and 516.5 eV, 524.5 and 517.4 eV in CNBG, respectively, while these peaks are located at higher values in BG (Fig. 2d). The three peaks at 533.1, 531.2, and 529.8 eV in CNBG (Fig. 2e) correspond to small molecules, bridging hydroxyls, and lattice oxygen,^34^ respectively, and together they are reduced relative to those in BG. In contrast, the N 1s spectra show an opposite trend (Fig. 2f). The binding energies of the C–N–H, N–(C)_3_, and C–N=C chemical bonds in CNBG are located at 404.1, 401.2, and 399.2 eV, respectively, which are higher values than those for pristine C_3_N_4_. These results ascertain that the Bi–O–N and V–O–N chemical bonds link the m-BiVO_4_/C_3_N_4_ heterostructure in the CNBG ternary composite. The electrons migrate from C_3_N_4_ to m-BiVO_4_ as a result of the Fermi level equilibrium upon hybridization.^26,35^

The C 1s spectra uncover whether the interfacial chemical interaction and the electron transfer are affected by the incorporation of C_3_N_4_ (Fig. S3†). The C 1s spectrum of graphene oxide (GO) was fitted into three peaks at 288.3 eV (C=O chemical bonds), 287.4 eV (C–O chemical bonds), and 285.3 eV (C=C chemical bonds).^36^ After *in situ* growth of m-BiVO4 on rGO, the C 1s spectrum of BG was divided into two peaks, at 285.8 eV (C–O chemical bonds) and 284.7 eV (C=C chemical bonds). Compared with BG, the C 1s spectrum of CNBG shows an additional peak at 288.6 eV (N=C–N chemical bonds).^37^ Conforming to the Bi 4f, V 2p, O 1s, and N 1s spectra results, the C 1s spectral analysis reconfirms that the C_3_N_4_ was incorporated into CNBG. In addition, there is an overall shift in the peaks over both BG and CNBG relative to GO, indicating the emergence of chemical bonds involving C atoms, *i.e.*, the V–C chemical bonds in the m-BiVO_4_/rGO Mott–Schottky heterostructure for both BG and CNBG (Fig. 2d). As a result, the higher Fermi level of rGO compared with that of m-BiVO_4_ results in an electron rearrangement,^38,39^ leading to a high conductivity region as well as downward band bending within m-BiVO_4_. When the m-BiVO_4_ absorbs visible light to generate electron–hole pairs, the rGO can accept electrons from m-BiVO_4_ through this high conductivity region in both BG and CNBG. A similar binding energy shift was found between black phosphorus (BP) and m-BiVO_4_/BP in the P 2p spectra in a previous study,^40^ indicating a strong interfacial chemical interaction between BP and m-BiVO_4_, as well as the electron transfer from m-BiVO_4_ to BP during photocatalytic reactions. Thus, the incorporated C_3_N_4_ makes no difference to both the V–C chemical bonds in CNBG and the electron transfer over the Mott–Schottky heterojunction.

When electrons transfer toward rGO, its degree of reduction determines the electron diffusion ability. In the C 1s spectra (Fig. S3†), the C=O and C–O chemical bonds of GO represent the OCGs, contributing to the *in situ* growth of m-BiVO_4_. For both BG and CNBG, the C–O peaks shifted more than the C=C peaks, causing shorter distances between the C–O and C=C peaks than for GO. This phenomenon indicates that some OCGs of GO turned into V–C chemical bonds in both BG and CNBG. Moreover, the absence of C=O peaks and diminished C–O peaks relative to GO indicate the removal of other OCGs, which agrees with the results of the Raman spectra. As the Raman bands range from 1000 to 2000 cm^−1^ (Fig. S4†), both D band (sp^3^ carbon defects) and G band (sp^2^ carbon atoms) are derived from the carbon network within the rGO. The lower intensity ratio (*I*_D_/*I*_G_) in BG (0.79) than in GO (1.37) reveals that the rGO is partially reduced in BG. Although a broad peak induced by disordered graphitic carbon–nitrogen vibrations overlaps with both the D band and G band in CNBG, a similar degree of reduction of rGO in CNBG can be speculated based on the lower *I*_D_/*I*_G_ in BG, as well as a facile composite process in CNBG. Therefore, Raman spectra and C 1s spectra elaborate an effective degree of reduction of rGO in both BG and CNBG, achieving a high conductivity and electron diffusion capacity for the carbon network after accepting electrons.

**Fig. 3:**
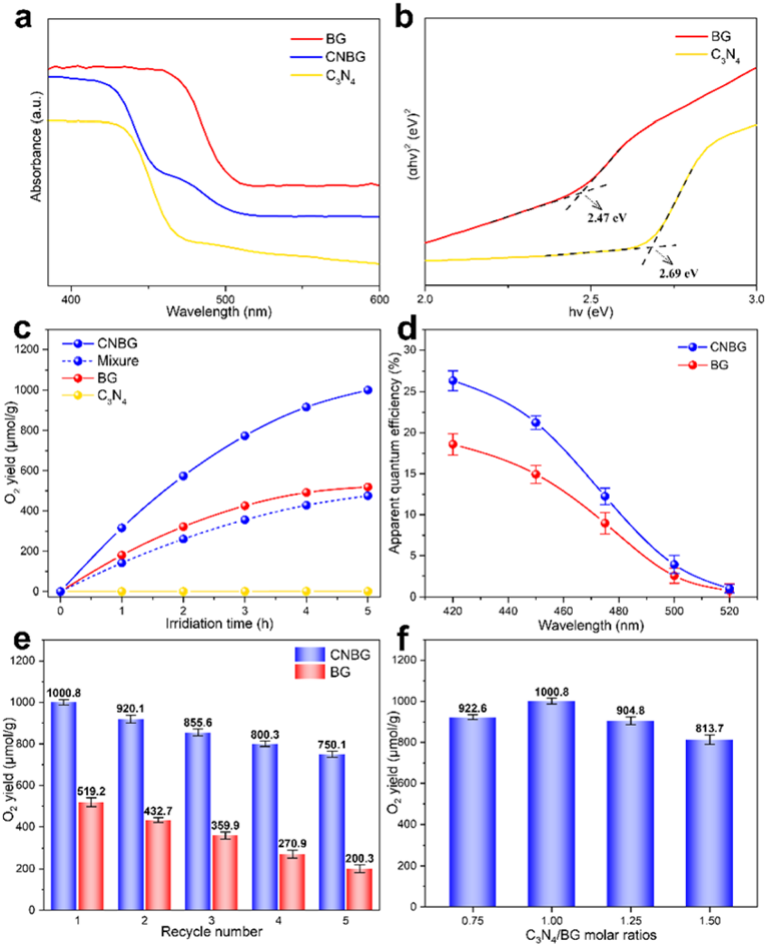
Characterization of the optical properties and photocatalytic performance for the C_3_N_4_, BG, and CNBG. (a) UV-vis absorption spectra. (b) (*αhv*)^2^*vs. hv* curves. Dashed lines mark baselines and tangents, the intersection value is the optical band gap. (c) Time course of O_2_ yield under visible light irradiation (*λ* > 420 nm). (d) AQE irradiated by monochromatic light. (e) Cycling stability tests and (f) the amount of O_2_ yield with different C_3_N_4_/BG molar ratios. Error bars represent the standard deviations of three independent measurements for the same sample.

An assessment of the energy band structure of the components is crucial to evaluate the origin of the charge carrier behavior. The photoresponse range of the photocatalysts was disclosed by UV-visible (UV-vis) absorption spectra (Fig. 3a), and pristine C_3_N_4_ and BG display discrete optical absorption edges attributed to their intrinsic band gap transitions. As for CNBG, there is a ladder-like optical absorption edge resulting from the overlapped band gap transitions of the C_3_N_4_ and BG components, which is consistent with the color change of the three photocatalysts (Fig. S5†). Furthermore, the CNBG exhibits similar absorption features to pristine C_3_N_4_ and BG, implying that the C_3_N_4_ linked with m-BiVO_4_ at the heterointerface is not incorporated into the m-BiVO_4_ lattice. This inference agrees with the XRD and Raman results. The optical band gap of C_3_N_4_ and BG can be quantitatively analyzed from Tauc plots (*i.e.*, the curve of (*αhv*)^*r*^*vs. hv*, where *r* = 2) for direct band gap semiconductors of m-BiVO_4_ (ref. 41) and C_3_N_4_.^42^ As shown in Fig. 3b, the optical band gap of BG is 2.47 eV and is smaller than that of C_3_N_4_ (2.69 eV), which is in harmony with their discrete optical absorption edges. Despite the larger optical band gap of C_3_N_4_ than of BG, both C_3_N_4_ and BG are able to realize visible light absorption. Hence, for the CNBG ternary composite, the m-BiVO_4_ component and C_3_N_4_ component concurrently trigger abundant photogenerated carriers upon visible light irradiation.

Besides the optical band gap, proper matching of the conduction band (CB) and valence band (VB) positions of the components with the redox potentials of photocatalytic reactions cannot be neglected. Measured by XPS VB spectra (Fig. S6a†), the VB positions of BG and C_3_N_4_ are +1.59 eV and +1.29 eV, respectively. Therefore, the VB position of the m-BiVO_4_ component in both BG and CNBG meets the thermodynamic demands for the PWO. Based on (*αhv*)^*r*^*vs. hv* curves and XPS VB spectra, the CB positions of BG and C_3_N_4_ were calculated as −0.88 eV and −1.40 eV, respectively. Hence, the approximate band positions of BG and C_3_N_4_ were drawn *vs.* a normal hydrogen electrode (NHE, pH = 7) in Fig. S6b.† Once the C_3_N_4_ was incorporated into the BG binary composite, a staggered energy band structure formed with the Fermi level of C_3_N_4_ much higher than that of m-BiVO_4_.^23,43^ The electrons of C_3_N_4_ would be transferred toward m-BiVO_4_*via* the Bi–O–N and V–O–N chemical bonds until the Fermi level equilibrium was reached, as corroborated by the Bi 4f, V 2p, O 1s, and N 1s spectral results. This spontaneous electron redistribution creates an electron depletion layer with upward band bending in C_3_N_4_, and an electron accumulation layer with downward band bending in m-BiVO_4_. Hence, an internal electric field (IEF) with a direction from C_3_N_4_ to m-BiVO_4_ appears at the heterointerface. Under visible light irradiation, both C_3_N_4_ and m-BiVO_4_ components became excited states with electron transition from the VB to the CB. The IEF can promote the transfer of electrons in the CB of m-BiVO_4_ across the heterointerface to consume holes in the VB of C_3_N_4_, *i.e.*, a Z-scheme electron-transfer pathway.^26,35^ In consequence, spatial separation of electron–hole pairs occurs in the heterointerface between m-BiVO_4_ and C_3_N_4_ in CNBG, preserving the strong reducing electrons in the CB of C_3_N_4_ and the strong oxidizing holes in the VB of m-BiVO_4_.

To illustrate the positive effect of the Z-scheme process on PWO activity, the activity was evaluated under visible light irradiation. Controlled tests revealed no O_2_ yield without photocatalysts or visible light. The CNBG exhibited an O_2_ yield of 1000.8 μmol g^−1^ (Fig. 3c), over 193% of that of BG, in the first 5 h, while a gap in the PWO performance between BG and a mixture of BG and C_3_N_4_ was small (within experimental error). Therefore, a strong interfacial chemical reaction, together with the Z-scheme process, explains the enhanced performance in CNBG. The pristine C_3_N_4_ showed scarce O_2_ yield owing to its weak PWO driving force induced by an unsuitable VB position. Also, the apparent quantum efficiency (AQE) was qualitatively analyzed with the help of different band-pass filters (Fig. 3d). The enhanced PWO performance of CNBG explains the maximum of 26.35% at 420 nm and is 1.42-fold higher than that of BG. As the light wavelength continues to increase, the gradually reduced AQE of both BG and CNBG validates that the PWO reaction is dependent on visible light absorption. However, the sharply decreased AQE of CNBG began at 475 nm, compared with BG. Since the m-BiVO_4_ component can be excited by visible light even up to 520 nm, whereas visible light beyond 475 nm cannot fully excite the C_3_N_4_ component (Fig. 3a), the photogenerated holes in the C_3_N_4_ component were deficient in annihilating photogenerated electrons in the m-BiVO_4_ component. This deduction supports the proposed Z-scheme pathway in CNBG. A comparison of the AQE of CNBG with the applied photocatalysts is provided in Table S1,† certifying that the Z-scheme heterostructure along with the Mott–Schottky heterostructure enables a superior PWO performance for CNBG relative to previous photocatalysts.

Except for the pristine C_3_N_4_ with scarce O_2_ yield, both BG and CNBG were collected to measure their durability. As shown in Fig. 3e, they both exhibited continuous O_2_ yield in each cycle. Considering that loss of photocatalyst in each cycle caused a decrease in performance, the CNBG still maintained over 374% of the performance of BG in the fifth cycle. XRD patterns and the TEM images confirm that the crystal phase and morphology of CNBG were sustained after the cycling tests (Fig. S7†), proving its reusability and photostability. To survey the impact of the incorporated amount of C_3_N_4_ on the PWO performance, CNBGs with different C_3_N_4_/BG molar ratios were prepared as reference samples (Fig. 3f). Evidently, the PWO performance continually decreased when the molar ratio exceeded 1.00. This downward trend is attributed to the excessive incorporation of C_3_N_4_ on the surface of the m-BiVO_4_ component causing shielding of the incident light,^44^ and thus inhibiting visible light absorption of the m-BiVO_4_ component and the Z-scheme electron transfer inside the CNBG.

**Fig. 4:**
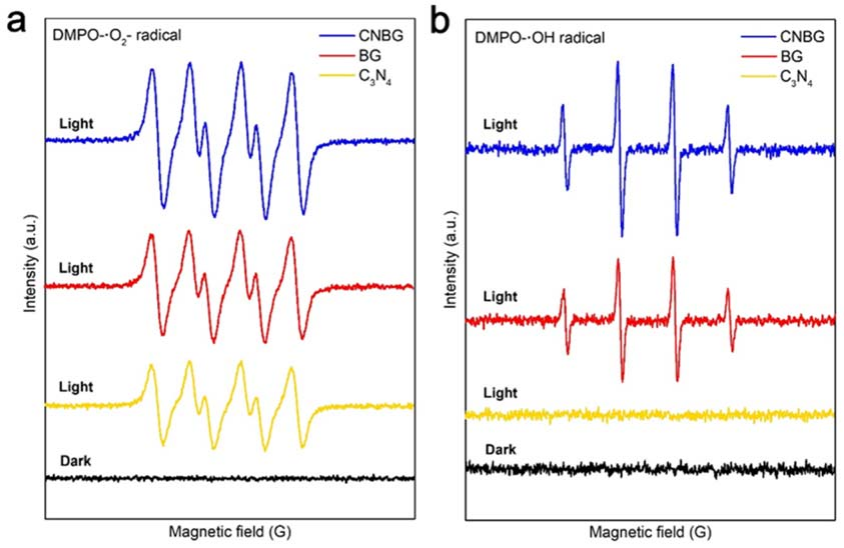
Characterization of reactive oxygen species for the C_3_N_4_, BG, and CNBG. ESR signals of (a) ·O^2−^ radicals and (b) ·OH radicals with and without visible light irradiation (*λ* > 420 nm).

Molecular oxygen activation measurements were performed to uncover the influence of the Z-scheme process on reactive oxygen species. Assisted by 5,5-dimethyl-l-pyrroline-*N*-oxide (DMPO), ·O^2−^ radicals and ·OH radicals were both detected. Electron spin resonance (ESR) signals from the DMPO^−^·O^2−^ adduct with light were found for pristine C_3_N_4_, BG, and CNBG (Fig. 4a), with ·O^2−^ radicals from O_2_ reduction by electrons; while peaks from the DMPO^−^·OH adduct with light were observed for BG and CNBG (Fig. 4b), with ·OH radicals originating from OH^−^/H_2_O oxidation by holes. By contrast, almost no DMPO^−^·OH adduct formed with light for pristine C_3_N_4_, which agrees with its scarce O_2_ yield under irradiation and was due to the VB position being too negative thermodynamically to oxidize OH^−^/H_2_O. Thereby ·OH radicals were mainly gathered in the m-BiVO_4_ component, while ·O^2−^ radicals were accumulated in the rGO component, or the C_3_N_4_ component in CNBG, supporting that the electron transfer follows the Mott–Schottky and Z-scheme pathway. Notably, the stronger signals from two adducts for CNBG than for BG affirm that more photogenerated electrons and holes were formed in CNBG, which can be ascribed to the increased spatial separation of the electron–hole pairs and the improved redox capability. Hence, based on the Mott–Schottky heterostructure of the BG binary composite, an additional Z-scheme heterostructure endows the CNBG ternary composite with formidable molecular oxygen activation ability, as well as superior PWO performance.

**Fig. 5:**
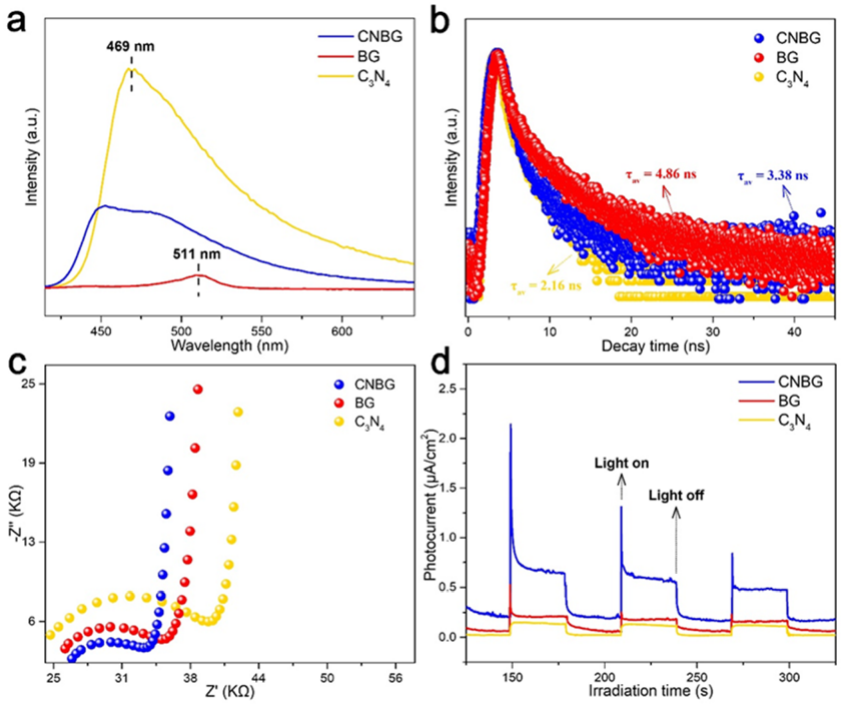
Exploration of the mechanism of the PWO reaction. (a) Steady-state PL emission spectra and (b) TRPL decay spectra. (c) EIS Nyquist plots and (d) transient photocurrent responses under visible light irradiation (*λ* > 420 nm).

Considering the prominent photocatalytic behavior of CNBG, we carried out controlled experiments to probe the reaction mechanism. To begin with photoluminescence (PL) spectra were obtained to survey the electron-transfer pathway (Fig. 5a). The pristine C_3_N_4_ and BG displayed two peaks centered at 469 and 511 nm, respectively, assigned to the intrinsic band gap emission. On the other hand, the CNBG showed a broad peak from 445 to 485 nm, which is not matched with the peaks of either pristine C_3_N_4_ or BG. The emission from the broad peak was assigned to the multiple heterointerface recombination of electrons in the CB of the m-BiVO_4_ component and of holes in the VB of the C_3_N_4_ component *via* Z-scheme electron transfer. In addition, the CNBG showed stronger fluorescence intensity than the BG. Although the high conductivity region that favors electron transfer exists over the Mott–Schottky heterostructure, the incorporated C_3_N_4_ can accelerate radiative recombination *via* the Z-scheme process, leading to fluorescence enhancement for CNBG. Moreover, time-resolved PL (TRPL) spectra were applied to further investigate the electron-transfer pathway (Fig. 5b). Based on the parameters from biexponential fitting (Table S2†), the fluorescence lifetime (*τ*_av_) value decreased from 4.86 ns for BG to 3.38 ns for CNBG. Despite a high conductivity region over the Mott–Schottky heterostructure, the decreased *τ*_av_ reconfirms an additional Z-scheme process that quenches low-energy electrons and holes in CNBG, in accord with the fluorescence enhancement described above. Therefore, the strong reducing electrons in the C_3_N_4_ component and strong oxidizing holes in the m-BiVO_4_ component are maintained with spatial separation, ultimately strengthening the photocatalytic behavior of the CNBG. As for the pristine C_3_N_4_, the strongest fluorescence intensity and the shortest *τ*_av_ imply its intrinsic high recombination rate of charge carriers without construction of a Z-scheme heterostructure.

Photoelectrochemical measurements were also employed to gain insight into the reaction mechanism. The electrochemical impedance spectroscopy (EIS) Nyquist plots were recorded under irradiation, and the arc radius was related to the interfacial transfer resistance (Fig. 5c). A smaller arc radius for CNBG than for BG verified the significance of an additional Z-scheme process in CNBG; namely, the spatial separation of strong reducing electrons and strong oxidizing holes in respective components raised the interfacial transfer efficiency. Measurements of transient photocurrent responses were conducted to evaluate the effect of the appended Z-scheme process on the photoelectron conversion efficiency (Fig. 5d). The photocurrent intensity of CNBG was 2.85 times that of BG, proving that more carriers are generated in double semiconductors and spatially separated *via* the Z-scheme process in CNBG. The almost unchanging photocurrent intensity with repeated on/off cycles illustrates the ideal photostability of CNBG. In addition to the results of the PL and TRPL spectra, the biggest arc radius and the lowest photocurrent intensity reconfirm the serious charge carrier recombination in pristine C_3_N_4_.

**Fig. 6:**
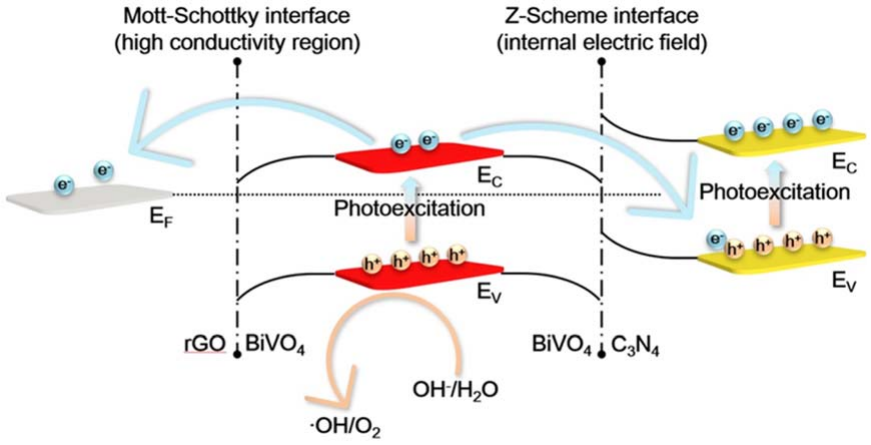
Schematic illustration of the PWO mechanism over CNBG. Here, EC, EV, and EF represent CB, VB, and Fermi levels, respectively.

Taking these results together, the PWO mechanism over CNBG is summarized as follows (Fig. 6): (1) on constructing the CNBG ternary composite, the sandwich-like hybrid architecture with chemical bonding fulfills the requirement for intimate face-to-face contact of the three components; (2) until the Fermi level equilibrium is reached, a high conductivity region exists at the m-BiVO_4_/rGO heterointerface and an IEF appears at the m-BiVO_4_/C_3_N_4_ heterointerface; (3) both C_3_N_4_ and m-BiVO_4_ components achieve excited states, with electron transition from the VB to the CB upon visible light irradiation; (4) the rGO accepts some photogenerated electrons from m-BiVO_4_ through a high conductivity region, and these electrons soon rapidly diffuse along the carbon network within rGO; (5) the IEF, meanwhile, promotes other photogenerated electrons in the CB of m-BiVO_4_, consuming the low-energy photogenerated holes in the VB of C_3_N_4_*via* the Z-scheme process; (6) the feasible spatial separation of electron–hole pairs sustains plenty of strong oxidizing holes in the VB of m-BiVO_4_, accomplishing the subsequent PWO reaction. As a consequence, an innovative strategy is proposed to develop multicomponent photocatalysts with dual-cascade charge-transfer pathways, aimed at high-efficiency artificial photosynthesis.

## Conclusions

Taking the unresolved surface recombination on the m-BiVO_4_ component in the BG binary composite into account, we mimicked natural photosynthesis to establish a new m-BiVO_4_/C_3_N_4_ Z-scheme heterostructure based on the m-BiVO_4_/rGO Mott–Schottky heterostructure. As a result, the face-contact CNBG ternary composite was elaborately constructed for PWO. In the heterointerface between m-BiVO_4_ and rGO, the rGO accepted photogenerated electrons from m-BiVO_4_ through a high conductivity region, achieving further electron diffusion along a carbon network. Noteworthy are the heterointerface between m-BiVO_4_ and C_3_N_4_, the IEF-promoted electrons in the CB of m-BiVO_4_ consuming holes in the VB of C_3_N_4_ upon irradiation, the spatial separation of the strong reducing electrons in the CB of C_3_N_4_, and the strong oxidizing holes in the VB of m-BiVO_4_. With these merits, the CNBG showed an O_2_ yield of 1000.8 μmol g^−1^, which is over 193% that of BG, together with an AQE of 26.35% at 420 nm, which is 1.42-fold higher than that of BG. In addition, the CNBG possessed excellent reusability and photostability. We have not only incorporated C_3_N_4_ into the BG binary composite to resolve carrier recombination on the bare surface of the m-BiVO_4_ component, but also unravelled the synergistic role of Mott–Schottky and Z-scheme heterostructures in a ternary composite, thus emulating the interfacial chemistry in artificial photosynthesis.

## Experimental section

### Materials synthesis

All chemicals were of analytical grade and were used without further purification. A GO sol–gel solution was prepared according to a previous study,^45^ and the pristine C_3_N_4_ was obtained by thermally exfoliating a mixture of pre-heated melamine and chloride according to a previous report.^46^

In a typical synthesis of m-BiVO_4_ grown *in situ* on rGO (BG), a GO sol–gel solution (containing 100 mg of GO) was dispersed in 85 mL of deionized water. Then, 631 mg BiCl_3_ and 400 mg cetyltrimethylammonium bromide were dissolved into the suspension by stirring for 4 h. Afterwards 234 mg NH_4_VO_3_ was dissolved into the suspension and stirred for 1 h. Soon after, excess 5 M aqueous ethanolamine was added into this suspension until pH 10, then the pH was mediated to 6.2 by adding 2 M hydrochloric acid. The solution was poured into an autoclave and heated at 160 °C for 12 h. After cooling, the product was centrifuged, washed, and collected by freeze-drying.

In a typical synthesis of CNBG, 70 mg of pristine C_3_N_4_ was dispersed in 80 mL of deionized water, and 80 mg of pristine BG was added into the mixed solution under stirring for 24 h. The product was centrifuged, washed, and collected by freeze-drying. Other reference samples were prepared under the same conditions but with different C_3_N_4_/BG molar ratios ranging from 0.75 to 1.50.

### Material characterization

Zeta potentials were measured in deionized water using a Zetasizer Nano ZS90. SEM images were recorded on a JEOL JSM-6360LV electron microscope. TEM and HRTEM were performed on a JEOL JEM-ARM200F electron microscope. The crystal phase was confirmed by X-ray diffraction using a Rigaku D/max 2550VL/PC system. Raman spectra were performed using a LabRAM HR system at 633 nm. XPS spectra were collected on an ESCALAB 250Xi instrument; the binding energies were referred to the C 1s peak from adventitious carbon. UV-vis absorption spectra were recorded with a PerkinElmer Lambda 750S UV-Vis-NIR spectrophotometer. The BET specific surface area was measured by using a nitrogen adsorption apparatus. The ESR spectra were acquired on a Bruker Elexsys E580 spectrometer with DMPO as the spin-trapping reagent. PL spectra were obtained on a Shimadzu RF-5301PC spectrophotometer at 365 nm. The fluorescence lifetime was quantified by the TRPL decay spectra on an Edinburgh FLS1000 spectrophotometer at 365 nm.

### Photoelectrochemical measurements

EIS spectra and transient photocurrent responses were obtained with 0.5 M Na_2_SO_4_ electrolytes in a standard three-electrode cell on a CHI 600E electrochemical workstation (Chenhua, China). Indium tin oxide (ITO) deposited with synthesized samples, platinum wires, and Ag/AgCl electrodes were utilized as the working electrodes, counter electrodes, and reference electrodes, respectively. To prepare the working electrode, 10 mg of sample and 500 μL of 5 wt% Nafion solution were dispersed in 5 mL of water–ethanol solution (*V*_water_/*V*_ethanol_ = 1 : 4) to form a homogeneous slurry, which was then spin-coated on 1.5 cm × 3.0 cm ITO and exposed to air. All working electrodes were prepared with the same conditions to guarantee the same loading. The EIS measurements were obtained under a constant potential of 0.6 V with irradiation, and the amplitude of the applied sine wave potential in each case was 5 mV. The transient photocurrent responses as the light switched on or off were measured at a constant potential of 0.6 V. The measurements were completed at room temperature. The potential in each case was 5 mV. The transient photocurrent responses as the light switched on or off were measured at a constant potential of 0.6 V. The measurements were completed at room temperature.

### Activity evaluations

The PWO activity of samples was tested with a PLS-SXE300 Labsolar-6A system (Perfectlight, China). A 30 mg portion of the sample was poured into 50 mL of 0.15 M Fe(NO_3_)_3_ solution in a Pyrex reaction vessel equipped with a quartz lid cooled by recirculating water. Soon after, the reaction setup was degassed to remove air, and the reaction solution was magnetically stirred and then irradiated under visible light. The O_2_ yield was recorded using a gas chromatograph (Panna G2090A), which was equipped with a thermal conductivity detector (column temperature at 60 °C, inlet temperature at 150 °C, test temperature at 125 °C). All peaks were examined against corresponding standards, and each test was conducted three times in parallel to obtain an average value.

### AQE measurements

The AQE was qualitatively analyzed under the same conditions as the activity evaluations, except for the insertion of band-pass filters (420, 450, 475, 500, 520 nm). The light intensity was measured at seven different points to obtain an average value using a PL-MW2000 optical power meter. The AQE was calculated using the following formula:^31^
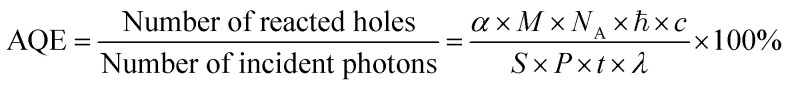
where *α* = 4 for the O_2_ yield, *M* is the amount of O_2_ molecule, *N*_A_ is Avogadro's constant, ℏ is Planck's constant, *c* is the speed of light, *S* is the irradiation area, *P* is the irradiation intensity, *t* is the irradiation time, and *λ* is the wavelength of the light.

## Conflicts of interest

There are no conflicts to declare.

## Supplementary Material

NA-005-D3NA00182B-s001
