# Supplementary material for: Nanoarchitectonics on Z-scheme and Mott–Schottky heterostructure for photocatalytic water oxidation via dual-cascade charge-transfer pathways
Source: Nanoscale Adv. 2023 May 10;5(12):3386–95. doi: 10.1039/d3na00182b (PMC10262966; doi:10.1039/d3na00182b)
Supplement: NA-005-D3NA00182B-s001 [file NA-005-D3NA00182B-s001.pdf]

Electronic Supplementary Material (ESI) for Nanoscale Advances.
This journal is © The Royal Society of Chemistry 2023

# Supporting Information

**Nanoarchitectonics on Z-scheme and Mott-Schottky heterostructure for photocatalytic water oxidation via dual cascade charge transfer pathways**

Yao Li, Siyuan Liu, Runlu Liu, Jian Pan, Xin Li, Jianyu Zhang, Xiaoxiao Zhang, Yixin Zhao, Dawei Wang, Hengdao Quan, Shenmin Zhu*

## 1. Supplementary Figures

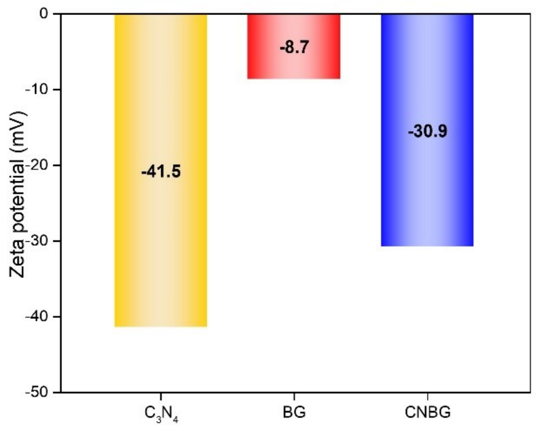


**Fig. S1. Zeta potentials of the $C_3N_4$, BG, and CNBG.**

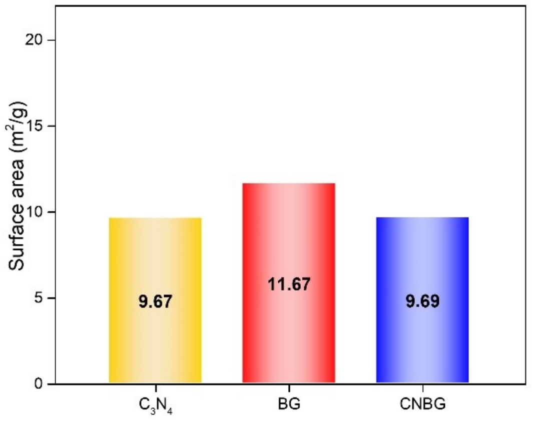


**Fig. S2. Specific surface areas of the $C_3N_4$, BG, and CNBG.**

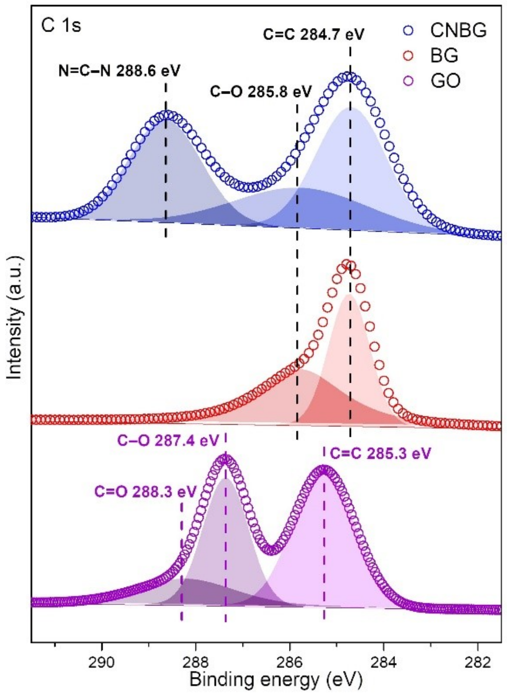


**Fig. S3. High-resolution C 1s XPS spectra of the GO, BG, and CNBG.**

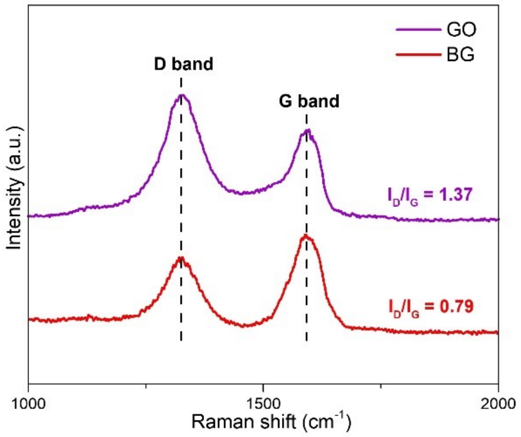


**Fig. S4. Raman spectra of the GO and BG.**

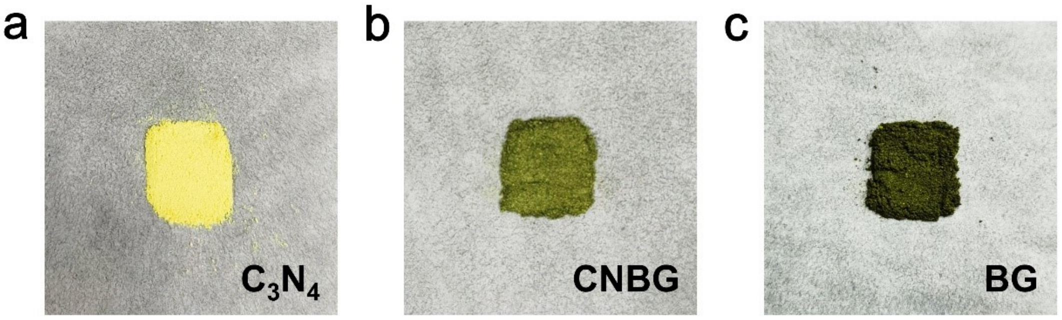


**Fig. S5. Digital photographs of the a) $C_3N_4$, b) CNBG, and c) BG.**

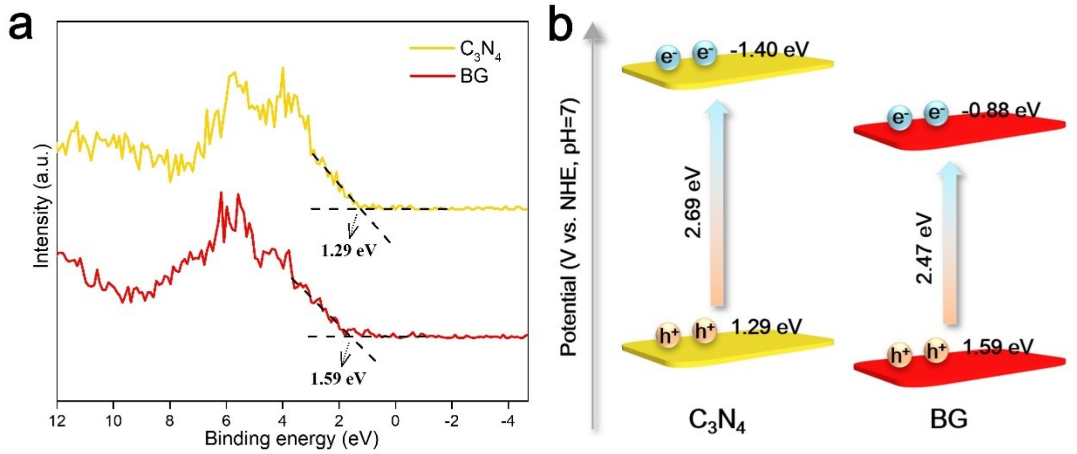


**Fig. S6. a) XPS valence band spectra of the $C_3N_4$ and BG. Dashed lines mark baselines and tangents, the intersection value is the valence band edge. b) Energy band structure diagrams of the $C_3N_4$ and BG.**

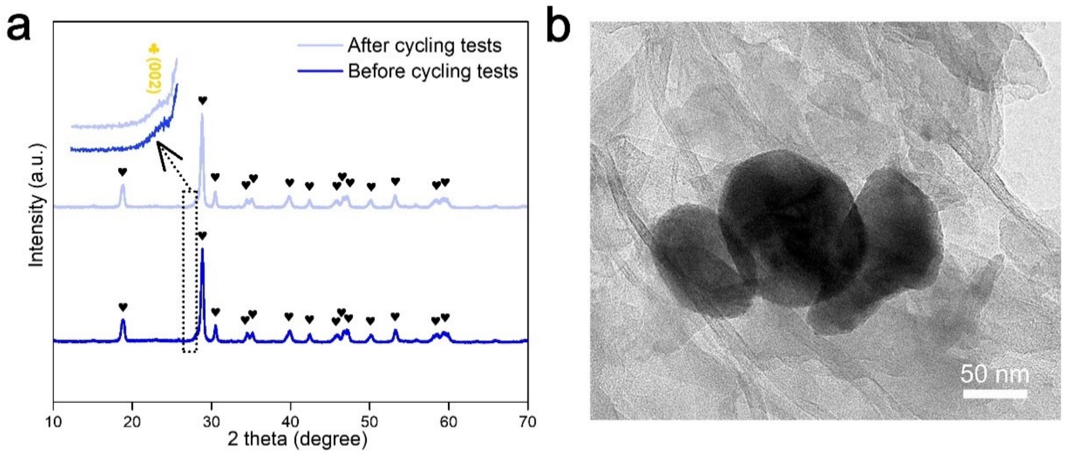


**Fig. S7. Characterizations for the CNBG before or after cycling tests. a) XRD patterns. b) The TEM image after cycling tests.**

## 2. Supplementary Tables

**Table S1. Comparison of apparent quantum efficiency (AQE) of the CNBG with reported photocatalysts.**

| Photocatalysts | AQE (%) | References |
|---|---|---|
| **CNBG** | **26.35 (420 nm)** | **This Work** |
| **$ZrO_2$/TaON/$BiVO_4$** | 12.3 (420 nm) | *Nature Communications.* **2022**, *13*, 484. |
| **$O_V$-rich BG** | 23.19 (420 nm) | *ACS Applied Materials & Interfaces.* **2022**, *14*, 12180-12192. |
| **$IrO_x$-am@$TiO_2$** | 7.82 (425 nm) | *ACS Catalysis.* **2020**, *10*, 8742-8750 |
| **30-facets $BiVO_4$ polyhedron** | 18.3 (430 nm) | *Advanced Materials.* **2018**, *30*, 1703119 |
| **Ultrapure $BiVO_4$/Graphene** | 19.8 (420 nm) | *Nanoscale.* **2020**, *12,* 14853-14862 |
| **$CoAl_2O_4$/g-$C_3N_4$** | 0.2 (420 nm) | *ACS Catalysis.* **2020**, *10*, 4960-4966 |
| **Au/$Al_2O_3$/$TiO_2$** | 1.6 (420 nm) | *Advanced Functional Materials.* **2020**, *281*, 2005688 |
| **ZnO/$BiVO_4$** | 5.0 (450 nm) | *Nano Energy.* **2018**, *51*, 764-773 |
| ***p*-BiOBr** | 1.6 (405 nm) | *Journal of the American Chemical Society.* **2022**, *144*, 3386-3397. |
| **$Zn_{0.2}$-PHI/PHI** | 3.6 (420 nm) | *ACS Catalysis.* **2021**, *11*, 13463-13471 |

**Table S2. Time-resolved PL decay curves were fitted by a biexponential function to calculate the fluorescence lifetime for the $C_3N_4$, BG, and CNBG.**

| Samples | $\tau_1$ (ns) | $A_1$ | $\tau_2$ (ns) | $A_2$ | $\tau_{av}$ (ns) | $\chi^2$ |
|---|---|---|---|---|---|---|
| **$C_3N_4$** | 0.87 | 1297.41 | 5.16 | 93.85 | 2.16 | 0.99 |
| **BG** | 0.88 | 774.42 | 6.11 | 353.62 | 4.86 | 0.99 |
| **CNBG** | 1.11 | 1122.03 | 13.17 | 21.89 | 3.38 | 0.99 |
